# Prospective study of dynamic whole-body 68Ga-DOTATOC-PET/CT acquisition in patients with well-differentiated neuroendocrine tumors

**DOI:** 10.1038/s41598-021-83965-9

**Published:** 2021-03-01

**Authors:** Philippe Thuillier, David Bourhis, Jean Philippe Metges, Romain Le Pennec, Karim Amrane, Ulrike Schick, Frédérique Blanc-Beguin, Simon Hennebicq, Pierre-Yves Salaun, Véronique Kerlan, Nicolas Karakatsanis, Ronan Abgral

**Affiliations:** 1 Department of Endocrinology, University Hospital of Brest, Boulevard Tanguy Prigent, 29609 Brest cedex, France; 2 EA GETBO 3878, University Hospital of Brest, Brest, France; 3 Department of Nuclear Medicine, University Hospital of Brest, Brest, France; 4 Department of Radiotherapy, University Hospital of Brest, Brest, France; 5 Department of Oncology, University Hospital of Brest, Brest, France; 6 Department of Radiology, Weil Cornell Medical College of Cornell University, New York, NY USA

**Keywords:** Cancer, Cancer imaging

## Abstract

To present the feasibility of a dynamic whole-body (DWB) ^68^Ga-DOTATOC-PET/CT acquisition in patients with well-differentiated neuroendocrine tumors (WD-NETs). Sixty-one patients who underwent a DWB ^68^Ga-DOTATOC-PET/CT for a histologically proven/highly suspected WD-NET were prospectively included. The acquisition consisted in single-bed dynamic acquisition centered on the heart, followed by the DWB and static acquisitions. For liver, spleen and tumor (1–5/patient), Ki values (in ml/min/100 ml) were calculated according to Patlak's analysis and tumor-to-liver (TLR-Ki) and tumor-to-spleen ratios (TSR-Ki) were recorded. Ki-based parameters were compared to static parameters (SUVmax/SUVmean, TLR/TSRmean, according to liver/spleen SUVmean), in the whole-cohort and according to the PET system (analog/digital). A correlation analysis between SUVmean/Ki was performed using linear and non-linear regressions. Ki-liver was not influenced by the PET system used, unlike SUVmax/SUVmean. The regression analysis showed a non-linear relation between Ki/SUVmean (R^2^ = 0.55,0.68 and 0.71 for liver, spleen and tumor uptake, respectively) and a linear relation between TLRmean/TLR-Ki (R^2^ = 0.75). These results were not affected by the PET system, on the contrary of the relation between TSRmean/TSR-Ki (R^2^ = 0.94 and 0.73 using linear and non-linear regressions in digital and analog systems, respectively). Our study is the first showing the feasibility of a DWB ^68^Ga-DOTATOC-PET/CT acquisition in WD-NETs.

## Introduction

Neuroendocrine tumors (NETs) are a group of rare tumors with a common embryological origin. NETs are characterized by a cellular over-expression of somatostatin receptors (SSTR) allowing the use of radio-labeled somatostatin analogs for diagnostic imaging or peptide-receptor radionuclide therapy (PRRT). Management of patients with NETs remains challenging and requires a multidisciplinary approach both for diagnosis, the histopathological characterization of the tumor and treatment strategy. For a long time, somatostatin receptor scintigraphy was used in the management of patients with well-differentiated NETs (WD-NETs)^1^. Currently, Gallium-68 DOTA-conjugated somatostatin receptor-targeting peptide (^68^Ga-DOTA-SST) positron-emission tomography computed tomography (PET/CT) is the mainstay for the diagnosis, staging and monitoring of WD-NETs^2,3^.

The capability of quantifying tracer uptake in PET is a real advantage, not only for tumor characterization (heterogeneity, cellular proliferation) but also for treatment management (response assessment, PRRT planning). Nevertheless, the actual quantification approach based on standardized uptake value (SUV) parameters extracted from a 3D static PET acquisition presents some limitations^4^. Several studies have already assessed the correlation between SUV-based parameters in ^68^Ga-DOTA-SST PET/CT and the SSTR expression of NETs, reporting different results according to the differentiation grade of tumors (i.e. WD-NETs and poorly differentiated neuroendocrine carcinomas (NEC)). Indeed, in a study including 21 NEC, a strong correlation (r = 0.89; p < 0.0001) was found between SSTR2 gene expression of tumor and SUVmax in ^68^Ga-DOTATOC PET/CT. On the contrary, an important overlap in SUVmax values according to SSTR expression grading system was reported in series concerning patients with WD-NETs, SUVmax not allowing to discriminate WD-NETs with weak, moderate or strong SSTR expression^5,6^. For example, Haug et al. fond only a weak correlation (r = 0.40, p < 0.05) in 27 patients with metastatic NETs^6^. Moreover, 3 out of 5 NETs without immunohistochemical SSTR expression showed a high uptake of ^68^Ga-DOTATATE. In addition to the diagnosis, this variance also appears to occur for the treatment strategy of WD-NETs. Indeed, a high SSTR expression demonstrated in ^68^Ga-DOTA-SST PET/CT is considered as an essential criterion before initiation of somatostatin analogs (SSA) or PRRT. However, few studies assessed the correlation between SUV-based parameters recorded on pretherapeutic ^68^Ga-DOTA-SST PET/CT and the outcome of patients treated by SSA. Only one cohort study, including 30 patients with WD-NET candidate to Octreotide LP, suggested a positive correlation between progression-free survival and SUVmax in ^68^Ga-DOTATATE-PET/CT^7^, but without significance in ROC analysis (area under the curve (AUC) = 0.647 with SUVmax threshold of 32, sensitivity and specificity of 75% and 64% respectively; p = 0.17). Therefore, the authors concluded that SUVmax value in ^68^Ga-DOTATATE-PET/CT could not be used alone but maybe integrated with a composite scoring system to predict treatment response to SSA^7^. Regarding studies assessing SUV-based parameters to predict the therapeutic response to PRRT, the results highlighted divergent conclusions. Thus, the choice of the most relevant parameter whether using the pretherapeutic SUV or the deltaSUV during PRRT treatment is not established. Once again, ROC analysis of SUV-based parameters alone remained often disappointing^8–11^.

Numerous studies have already demonstrated the advantage of quantifying 18-fluorodesoxyglucose (^18^FDG) uptake based on compartmental modeling approaches using a 4D dynamic PET/CT acquisition to improve both tumor characterization and treatment response assessment in comparison with 3D static acquisition^12^. Nevertheless, only few studies assessed the usefulness of dynamic acquisition in ^68^Ga-DOTA-SST PET/CT in patients with NETs^13–16^. In a small cohort of 10 patients, Velikyan et al. studied the correlation between the Net Uptake Rate (Ki) and the SUV for both ^68^Ga-DOTATOC and ^68^Ga-DOTATATE tracers. Their results showed no linear correlation between SUVmean and Ki, SUVmean not increasing when Ki values exceeded 0.2 ml/min/ml. The authors suggested that the SUV did not correctly reflect the density of receptors for highly-expressed SSTR tumors and could lead to an underestimation of their expression in some cases^13^. However, these studies proposed a one-step dynamic acquisition, limiting the analysis to a sole region of the body and thus constituting a limitation in the evaluation of metastatic multifocal cancers mainly prevalent in NETs^17^. Recently, multi-step or flow motion acquisition protocols have been developed in the latest generations of PET machines, making possible to perform a dynamic whole-body (DWB) analysis in a single scan^18–22^. To our knowledge, no study has already assessed the interest of a DWB ^68^Ga-DOTATOC-PET/CT acquisition in patients with NETs.

The objectives of this study are: (i) to present the feasibility of a DWB ^68^Ga-DOTATOC-PET/CT acquisition in two PET systems (i.e. analog versus digital) in a large population of patients with WD-NETs; (ii) to describe derivated Ki parameters within tumor lesions and healthy organs; and (iii) to compare them with the corresponding SUV parameters, according to the cohort characteristics.

## Materials and methods

This is a prospective monocentric study ancillary to the GAPET-NET trial (NCT03576040).

### Population

#### Inclusion criteria

Inclusion criteria were as follows: patients ≥ 18 years old, undergoing a DWB ^68^Ga-DOTATOC-PET/CT acquisition for the staging or restaging of a histologically proven or highly suspected WD-NETs.

Exclusion criteria were as follows: minor patient, other tumor types, tracer contraindication (pregnancy, breastfeeding), refusal to participate.

The protocol was approved by the institutional medical ethics committee of Brest (29BRC17.0036). Informed consent was obtained from all the patients to participate in the study.

### Patients characteristics

Sixty-one patients (32M/29F) with a mean age of 59.3 ± 15.6 years were included between July 2018 and July 2019. The clinical characteristics of the population are presented in Table 1. The most frequent primary tumor origin (34/61 = 55.7%) was the foregut, including 30 patients with a pancreatic NET. Fourteen (23%) patients had a secretory syndrome and 37 (61%) presented a metastatic disease. Histopathological grading was not possible in 14 cases for the following reasons: typical imaging of NET in 9 cases (no cytological exploration in 7 patients with known MEN-1; non-contributory endoscopic ultrasound fine-needle aspiration in 2 patients with small size pancreatic tumors) and no Ki67 immunohistochemistry of a proven WD-NET documented in 5 cases (not carried out in routine practice on the date of diagnosis). Among the 47 remaining patients, 29 (63%) had a grade 2 WD-NET. The median Ki-67 value was 5% [IQR, 2–10]. Nineteen (31%) patients were treated with SSA therapy at the time of the PET/CT. The ^68^Ga-DOTATOC-PET/CT was performed for the stating or the restaging of the disease in 31 (50.8%) and 30 (49.2%) cases, respectively.

**Table 1:** Characteristics of patients.

Characteristics	Patients (n = 61)
**Age (years), mean ± Sd**	59.3 ± 15.6
**Sex (male/female), n**	32/29
**MEN1, n (%)**	
Yes	7 (11.5)
No	52 (85.2)
In progress	2 (3.3)
**Primary location, n (%)**	
Foregut	34 (55.7)
Lung	3 (5.0)
Pancreas	30 (49.1)
Duodenum	1 (1.6)
Midgut	21 (34.4)
Small bowel	18 (29.5)
Appendix	1 (1.6)
Caecum	2 (3.3)
Hindgut	2 (3.3)
Rectum	2 (3.3)
Other	1 (1.6)
Unknown	3 (5.0)
**Hormonal syndrome, n (%)**	
No	47 (77)
Yes	14 (23)
Carcinoid syndrome	6 (9.8)
Gastrinoma	3 (5.0)
Insulinoma	2 (3.3)
Glucagonoma	1 (1.6)
VIPoma	1 (1.6)
Hypercalcemia	1 (1.6)
**TNM staging, n (%)**	
T (1/2/3/4/X^a^)	5/10/18/6/22
N (0/1/X^a^)	16/32/13
M (0/1/X^a^)	24/37/0
**Grade, n (%)**	
1	18(38.3)
2	29(61.7)
Unknown	
**Ki67, n (%; median [IQR])**	5 [2–10]
**Past treatment n (%)**	
Surgery	29 (47.5)
Radiotherapy	1 (1.6)
Systemic treatment	7 (11.5)
Somatostatin analogs	3 (4.9)
Chemotherapy	5 (8.2)
Targeted therapy	1 (1.6)
Locoregional treatment	3 (4.9)
PRRT	0 (0)
**Ongoing systemic treatment n (%)**	
Systemic treatment	20 (32.8)
Somatostatin analogs	19 (31.2)
Chemotherapy	2 (3.3)
Targeted therapy	2 (3.3)
PRRT	2 (3.3)

^a^Not available or unknown.

### PET/CT imaging

All images were acquired on two different Biograph (Siemens©, Erlangen, Germany) PET machines: a mCT system until October 2018; and after a digital Vision system (Siemens©, Erlangen, Germany). The DWB PET were acquired on the mCT and the Vision systems in 39 (74%) and 22 (36%) cases respectively. The mean injected tracer dose was of 194.1 ± 42.4 MBq (range: 122–298) and the mean injected tracer dose (MBq) per kg was of 2.66 ± 0.32 (range: 2.02 – 3.54).

CT data were acquired first after injection of intravenous iodine contrast agent (1.5 mL/kg), unless contraindicated. The CT consisted of a 64-slice multidetector-row spiral scanner with a transverse field of view of 700 mm. The CT parameters were: collimation of 16 × 1.2 mm, pitch = 1, tube voltage and exposure automatically regulated (CarekV, CareDose 4D) with 120 kV and 80 Qref mAs as basic parameters. The CT images were reconstructed with an iterative method (SAFIRE, strength 5).

PET images were then acquired immediately after the injection of ^68^Ga-DOTATOC prepared with gallium-68 eluted with a Galliapharm generator (Eckert and Ziegler©, Berlin, Germany) and DOTATOC kits purchased from AAA (Advanced Accelerator Applications©, Saint Genis Pouilly, France).

PET data were reconstructed without and with attenuation correction using an iterative reconstruction algorithm (OSEM 3D) with “time of flight” (ToF) and point spread function (PSF) correction (Siemens© TrueX). PET images were corrected for random coincidence, scatter and attenuation using CT data; and smoothed using a Gaussian filter (2 mm wide at mid-height). The sizes of the transaxial reconstruction matrix were respectively 200 × 200 (voxel size = 4.07 × 4.07 × 2 mm) and 440 × 440 (voxel size = 1.65 × 1.65 × 1.65 mm) on the mCT and the Vision systems.

#### Dynamic whole-body PET protocol

**Figure 1:**
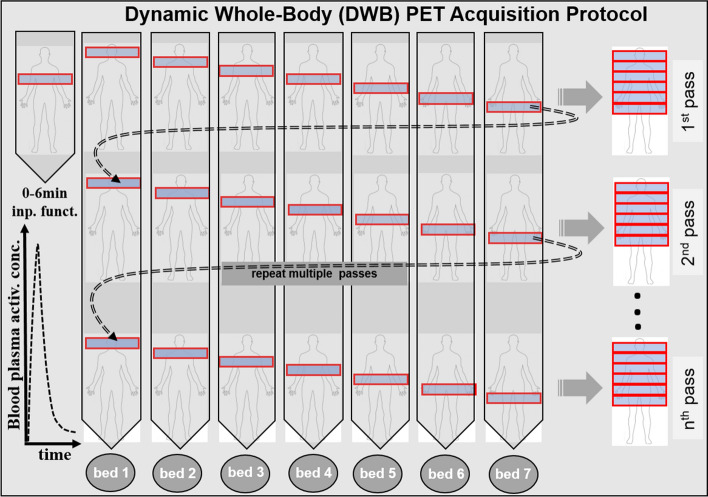
Flow chart illustrating the sequence of all dynamic bed frames, as acquired with the step and-shoot mode during the 2nd phase of the DWB acquisition: 6 unidirectional (cranio-caudal) WB passes are acquired, each comprised of 7 beds of equal scan duration**.** The figure was drawn using 2016 Microsoft PowerPoint application (Microsoft, Germond, WA, United states).

The DWB PET acquisition was performed (Fig. 1, DWB scan protocol) according to the methodology previously described by Karakatsanis et al.^20^.

A single dynamic cardiac-bed (DCB) position acquisition was followed by a DWB cranio-caudal step and shoot multi-pass acquisition : on the mCT system = 6-min DCB (12 images × 10 s, 12 images × 20 s) + 42-min DWB (7 passes, 6 min/pass) (Fig. 1); on the Vision system = 6-min DCB (12 images × 5 s, 6 images × 10 s, 8 images × 30 s) + 54-min DWB (9 passes, 6 min/pass).

#### Static images

On the mCT system, the DWB protocol was followed by a conventional static acquisition. The PET data were then acquired after the DWB acquisition using a whole-body protocol (2 min per step, 200 × 200 matrix) and were reconstructed using an ordered subsets expectation–maximization (OSEM) algorithm (TrueX = PSF (point spread function) + TOF (time of flight) OSEM-3D with 4 × 4 × 2 mm voxels.

On the Vision system, the static images were generated by adding the PET raw data from the last 2 dynamic passes (8th and 9th)^20,23^.

### PET/CT analysis

Quantification of physiological and tumoral tracer uptakes have been analyzed on both static and dynamic PET images.

#### Static parameters

Circular regions of interest (ROI) were drawn over a non-invaded part of the liver and spleen organs to record SUVmax and SUVmean. For that, a 3-cm and a 1-cm diameter circular ROIs in the right hepatic lobe and in the spleen were respectively used, as previously recommended^24^.

Spherical volumes of interest (VOI) were drawn over each tumor lesions, corresponding to primary location and/or lymph nodes and/or metastases (up to 5 per patient). Lesions were segmented using a fixed SUV threshold method delineating a 3D contour around voxels equal to or greater than 40% of SUVmax of the lesion. SUVmax and SUVmean, defined respectively as the the maximal and mean SUV in the segmented lesion, were recorded.

Mean tumor-to-liver ratio (TLRmean) and tumor-to-spleen ratio (TSRmean), defined as the tumor SUVmean corrected respectively for the SUVmean in the liver and the spleen, were calculated.

#### Dynamic parameters

Mean net uptake rate (Ki) values (in ml/min/100 ml) for physiological (Ki-liver, Ki-spleen) and tumor uptakes (Ki-tumor) were recorded. Tumor-to-liver ratio (TLR-Ki) and tumor-to-spleen ratios (TSR-Ki), defined as the Ki-tumor corrected for the Ki-liver and the Ki-spleen respectively, were calculated.

##### Input function

As previously described^13^, the total radioactivity concentration in the arterial plasma was used to model the input function (IF). A VOI of 1 cm was drawn as close as possible at the center of the left ventricle and away from the myocardium to mitigate any partial volume effects^25^ for each image of the DCB. The VOI was then projected on the DWB images to generate the IF. Theoretically, an arterial blood sample is required to obtain an IF, but several studies have shown that it can be estimated only from image data^16,26,27^.

**Figure 2:**
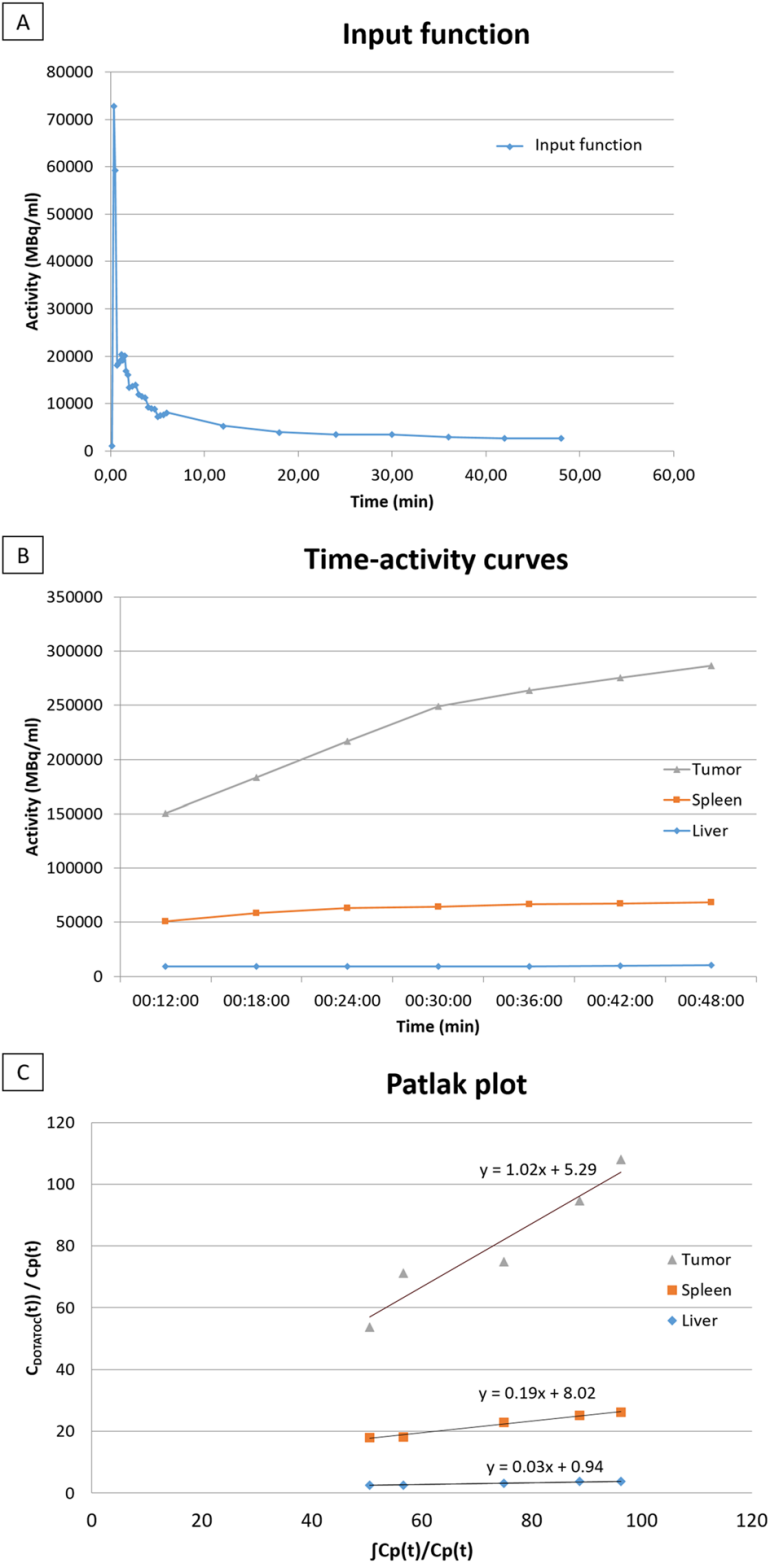
Example of dynamic acquisition data processing in a patient with a highly DOTATOC-avid pancreatic tumor (SUVmax = 159.54 SUVmean 98.29 and Ki = 102.41 ml/min/100 ml) (Top: Input function; Middle: Time-activity curve; Bottom: Patlak Plot).

An example of IF is illustrated in the Fig. 2.

##### Ki extraction

On the mCT system, Ki values were calculated on the Excel software (Microsoft, Germond, WA, United states) from a Patlak graphical analysis of the time-activity curves (TAC). To compute TAC, we applied liver and spleen ROIs and lesions VOIs previously generate on static images on each pass of the DWB acquisition. Patlak plots were then calculated from the 3rd to 7th passes of the DWB acquisition (18–48 min). Ki values were finally deduced by linear regression of the following equation^28^:

1 $$\frac{{\mathrm{C}}_{\mathrm{DOTATOC}}\left(\mathrm{t}\right)}{\mathrm{Cp}\left(\mathrm{t}\right)}=\mathrm{ Ki}\frac{{\int }_{0}^{\mathrm{t}}\mathrm{Cp}\left(\mathrm{t}\right)\mathrm{dt}}{\mathrm{Cp}\left(\mathrm{t}\right)}+\mathrm{Vp}$$

V_p_: volume fraction of plasma in the VOI, C_p_: arterial plasma blood concentration and C_DOTATOC_: tissue concentration.

An example of TAC and Patlak plot is shown in Fig. 2.

On the Vision system, Ki values were automatically generated on reconstructed Patlak parametric images^21,22^.

### Statistical analysis

#### Relation between static and dynamic parameters in the whole cohort and according to PET system

A correlation analysis between static and dynamic parameters was also performed. We assessed the evolution of the SUVmean as a function of the Ki according to two fitting models proposed in the literature: linear regression^16^ (SUVmean = A*Ki) and non-linear regression using an inverse hyperbolic fit (SUVmean = Ki/(A*Ki + B)). A coefficient of determination R^2^ was used to assess the degree at which each model can predict the variance of the measured SUVmean scores using the following definition :

2 $${R}^{2}=1- \frac{{SS}_{res}}{{SS}_{tot}}$$

SS_tot_ is the total sum of squares, which is defined as the sum of squared differences of the measured SUVmean scores to their mean, and is proportional to the total variance of the SUVmean data. On the other hand, SS_res_ is the residual sum of squares expressing the variance of the model’s errors and is defined as the sum of the squared differences (fit residuals) between the fitted SUVmean scores, as predicted from the measured Ki scores according to each fitted model, and the respective measured SUVmean scores. According to the definition, the coefficient of determination can have values from − ∞ to 1, thus it can be negative despite its squared notation that may suggest otherwise. A value of 1 suggests the model’s ability to exactly predict the observed SUVmean data variance, a value of 0 denotes ability to predict only the mean of the SUVmean scores, while a negative value implies prediction of scores with larger fitting error variance than the variance of the SUVmean data.

Based on previous studies suggesting that SUV values do not increase when Ki values exceeded 20 ml/min/100 ml^13,15^, the median value of Ki/SUVmean ratio (KSR) as Ki increased according to the following Ki intervalls (0–20, 20.1–40, 40.1–60, and > 60 ml/min/100 ml) were first compared using a Kruskal–Wallis test.

To assess the impact of the digital detection on the model on the relation between static and dynamic parameters, the median values of physiological/tumoral static (SUVmax, SUVmean) and dynamic (Ki) parameters according to the type of PET system (analog versus digital) were then compared. A correlation analysis using linear or non-linear regression according to the results obtained in the whole cohort was also performed.

#### Static and dynamic parameters according to cohort characteristics: exploratory analysis

Static and dynamic parameters were also compared in patients according to their characteristics : histologic grade (G1 vs. G2), Ki67 (< 5% vs. ≥ 5%), primary tumor site (pancreatic versus others) ongoing SSA treatment. A correlation analysis in subgroup of patients with ongoing SSA treatment (SSA + group) versus no SSA treatment (SSA- group) was finally performed to assess the influence of a potential competition between the tracer and the treatment on SSTR.

The significance level of the p-value was 0.05. All statistical analyses were performed using XLStat 2019 (Addinsoft©, Paris, France) and Excel softwares.

### Ethics approval and consent to participate

The study was conducted in accordance with the Declaration of Helsinki, Good Clinical Practice, and relevant French regulations regarding ethics and data protection. Informed consent was obtained from all the patients to participate in the study. The study (NCT03576040) was approved by our university hospital’s institutional review board (29BRC17.0036).

### Consent for publication

All authors contributed to drawing up the manuscript and approved this version.

## Results

A total of 175 lesions, comprising 1, 2, 3, 4 and 5 lesions in respectively 22 (36%), 7 (11.5%), 8 (13.1%), 5 (8.2%) and 19 (31.2%) patients, were segmented. Of the 61 patients, 48 had a sufficient part of healthy liver tissue allowing an analysis of physiological hepatic uptake. The study of physiological spleen uptake was not possible in 6 patients who had previously undergone a splenectomy.

### Static and dynamic parameters in the whole cohort and according to PET system

The median values of the physiological, tumoral and ratio in static and dynamic ^68^Ga-DOTATOC-PET/CT parameters are shown in Table 2. The median values of SUVmax-liver and SUVmean-liver were respectively 7.7 [IQR, 6.4–9.2] and 4.8 [IQR, 3.8–5.7]. The median values of SUVmax-spleen and SUVmean-spleen were respectively 25.7 [IQR, 15.4–33.3] and 20.5 [IQR, 11.8–26.5]. The median values of Ki-liver and Ki-spleen were respectively 2.5 ml/min/100 ml [IQR, 1.5–3.2] and 10.6 ml/min/100 ml [IQR, 6.7–14.9]. The median values of SUVmax-tumor and SUVmean-tumor were respectively 39 [IQR, 22.9–60.1] and 22.9 [IQR, 14.8–38.7]. The median values of Ki-tumor was 16.9 ml/min/100 ml [IQR, 10.1–30.0]. The median values of TLR-Ki and TSR-Ki were higher than TLRmean and TSRmean (9.2 [IQR, 6.1–13.1] versus 5.3 [IQR, 3.3–7.2]; and 1.9 [IQR, 1–3] versus 1.5 [IQR, 0.8–2.5] (p < 0.001 both), respectively).

**Table 2:** Median values of static and dynamic parameters.

Parameters	Median [IQR]
**Physiological**	
Liver	
SUVmax	7.7 [6.4–9.2]
SUVmean	4.8 [3.8–5.7]
Ki (in ml/min/100 ml)	2.5 [1.5–3.2]
Spleen	
SUVmax	25.7 [15.4–33.3]
SUVmean	20.5 [11.8–26.5]
Ki (in ml/min/100 ml)	10.6 [6.7–14.9]
**Tumoral**	
SUVmax	39.0 [22.9–60.1]
SUVmean	22.9 [14.8–38.7]
Ki (in ml/min/100 ml)	16.9 [10.1–30]
**Ratios**	
TLRmean	5.3 [3.3–7.2]
TSRmean	1.5 [0.8–2.5]
TLR-Ki	9.2 [6.1–13.1]
TSR-Ki	1.9 [1.0–3.0]

**Figure 3:**
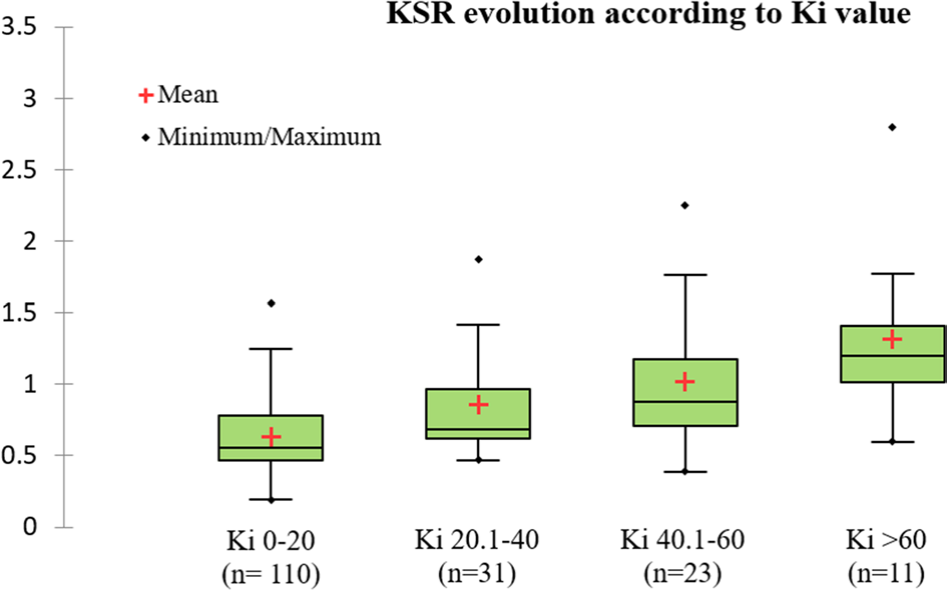
KSR evolution according to Ki intervalls (0–20, 20.1–40, 40.1–60, and > 60 ml/min/100 ml). KSR was defined as the ratio between Ki-tumor to SUVmean-tumor (KSR).

The median value of KSR was 0.65 [IQR, 0.52–0.93] with a wide range of values from 0.19 to 2.79. The KSR median value increased significantly when Ki also increased (p < 0.001). (Fig. 3).

According to PET system, the median value of SUVmax-liver was significantly higher in the Vision PET system group than in the mCT PET system group (8.3 [IQR, 7.6–9.8] vs 7.4 [IQR, 6.1–8.8], p = 0.027) (Table 3). There was no difference between the others static and dynamic parameters according to the 2 PET systems (Tables 3, 4).

**Table 3:** Median values of physiological parameters according to PET system and SSA therapy.

Parameters	SUVmax (median [IQR])	SUVmean (median [IQR])	Ki (in ml/min/100 ml) (median [IQR])
**Liver**			
PET system			
mCT	7.4 [6.1–8.8]	4.8 [3.3–5.3]	2.4 [1.4–3.0]
Vision	8.3 [7.6–9.8]	5.5 [4.3–6.1]	2.7 [1.7–3.3]
p-value	**0.027**	0.060	0.357
SSA therapy			
SSA+	4.7 [4.4–6.9]	2.7 [2.4–3.7]	1.3 [0.8–1.5]
SSA−	8.1 [7.3–9.3]	5.0 [4.4–5.9]	2.7 [2.2–3.3]
p-value	**0.001**	**0.002**	** < 0.001**
**Spleen**			
PET system			
mCT	24.8 [14.9–32.4]	19.9 [11.8–25.5]	10.4 [6.7–15.8]
Vision	26.6 [18.4–34.5]	21.3 [13.4–27.5]	11.3 [6.0–14.4]
p-value	0.687	0.605	0.769
SSA therapy			
SSA+	12.8 [10.5–15.5]	9.9 [8.1–11.4]	5.7 [3.7–6.8]
SSA−	29.6 [24.8–34.8]	24.3 [19.9–28]	13.9 [10.3–17.9]
p-value	** < 0.001**	** < 0.001**	** < 0.001**

Bold characters corresponded to p value <0.05

**Table 4:** Median values of tumoral parameters according to the cohort characteristics.

Parameters	SUVmax (median [IQR])	SUVmean (median [IQR])	Ki (in ml/min/100 ml) (median [IQR])
**Tumor**			
PET system			
mCT (n = 39)	36.5 [22.8–58.4]	22.1 [14.2–39.1]	17.9 [10.6–32.9]
Vision (n = 22)	42.3 [32.3–64.6]	25.3 [19.2–38.1]	15.3 [10.0–24.0]
p-value	0.599	0.702	0.323
Embryologic origin			
Foregut (n = 34)	50.6 [36.3–85.1]	30.6 [21.2–49.4]	26.3 [13.4–34.7]
Midgut (n = 21)	22.4 [17.9–43.6]	13.7 [10.5–26.4]	9.6 [5.0–16.1]
p-value	** < 0.001**	** < 0.001**	** < 0.001**
Histologic grade			
1 (n = 18)	44.2 [27.3–58.3]	26.7 [16.2–39.3]	19.0 [9.8–32.0]
2 (n = 29)	36.5 [22.7–53.7]	22.1 [13.7–31.0]	16.4 [9.9–24.5]
p-value	0.511	0.525	0.620
SSA therapy			
SSA+ (n = 19)	33.2 [22.5–44.2]	20.2 [13–26.7]	12.6 [8.4–17.6]
SSA− (n = 42)	43.1 [26.6–71.2]	27.8 [16–40.3]	20.1 [11.7–33.0]
p-value	0.083	0.0977	**0.027**

Bold characters corresponded to p value <0.05

#### Correlation between static and dynamic parameters

##### Physiological and tumor uptake

**Figure 4:**
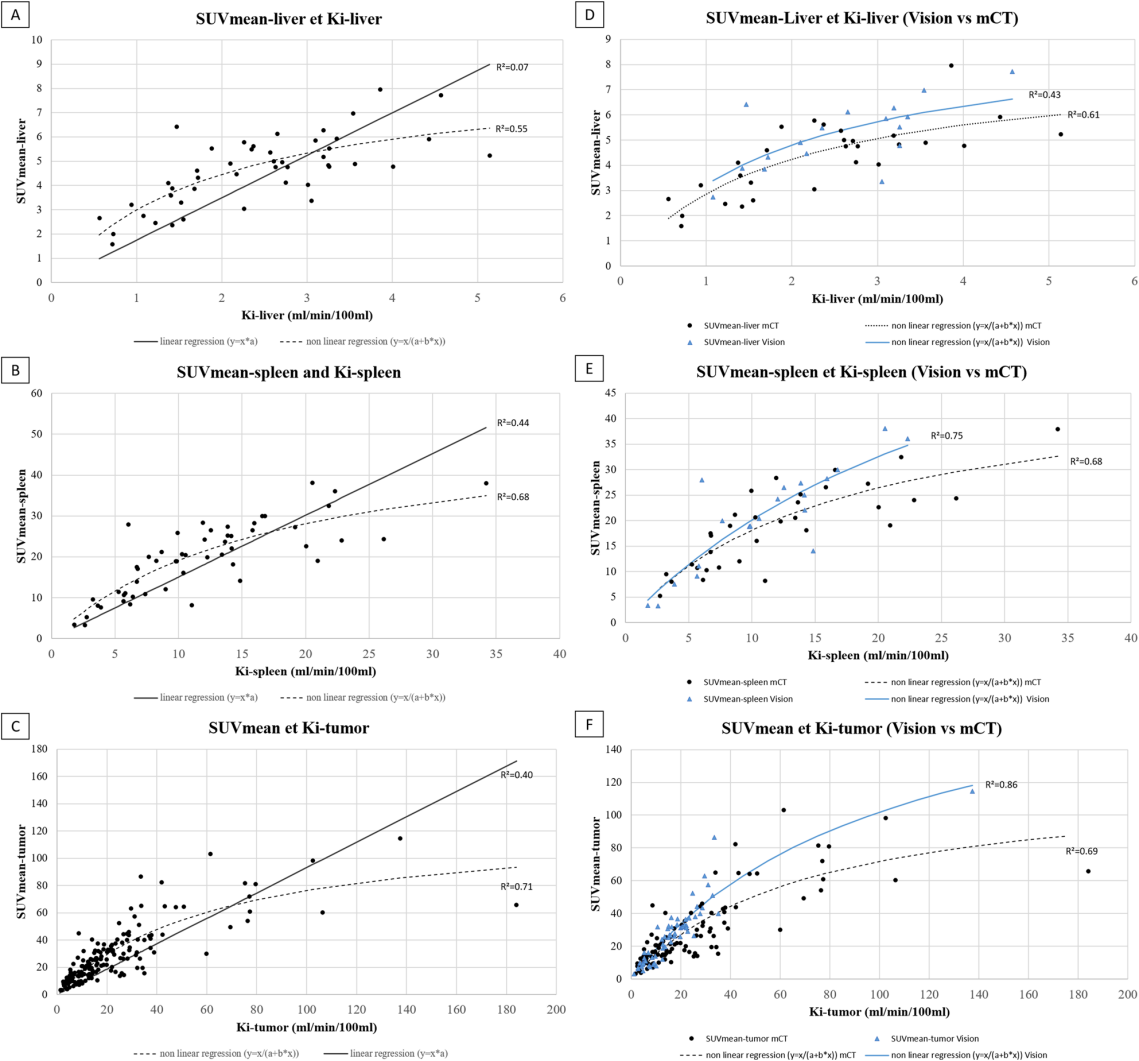
Correlation between SUVmean and Ki for physiological and tumoral uptakes in the whole cohort and according to PET system.

The relation between Ki and SUVmean was non-linear for liver and spleen uptake (R^2^ = 0.55 and 0.68 versus 0.07 and 0.44 in non-linear versus linear regressions, respectively (Fig. 4A,B; Table 5). According to PET system, R^2^ were 0.43 and 0.61 in PET Vision and mCT system in non-linear regression, respectively (Fig. 4D) for liver uptake. For spleen uptake, R^2^ were 0.75 and 0.68 in PET Vision and mCT system in non-linear regression, respectively (Fig. 4E).

**Table 5:** Coefficient of determination (R^2^) between static and dynamic parameters using a linear and a non-linear regression.

Parameters	Linear regression	Non-linear regression
**Physiological**		
Liver		
Ki-mean/SUVmean	0.07	0.55
Spleen		
Ki-mean/SUVmean	0.44	0.68
**Tumoral**		
Ki-mean/SUVmean	0.40	0.71
**Ratios**		
TLR-Ki/TLRmean	0.75	0.79
TSR-Ki/TSRmean	0.63	0.76

##### Tumor uptake

The relation between Ki-tumor and SUVmean-tumor was non-linear (R^2^ = 0.71 versus 0.40 in non-linear versus linear regressions, respectively; Fig. 4C) (Table 5). According to PET system, R^2^ were 0.86 and 0.69 in PET Vision and mCT system in non-linear regression, respectively (Fig. 4F);

##### TLR and TSR ratios

**Figure 5:**
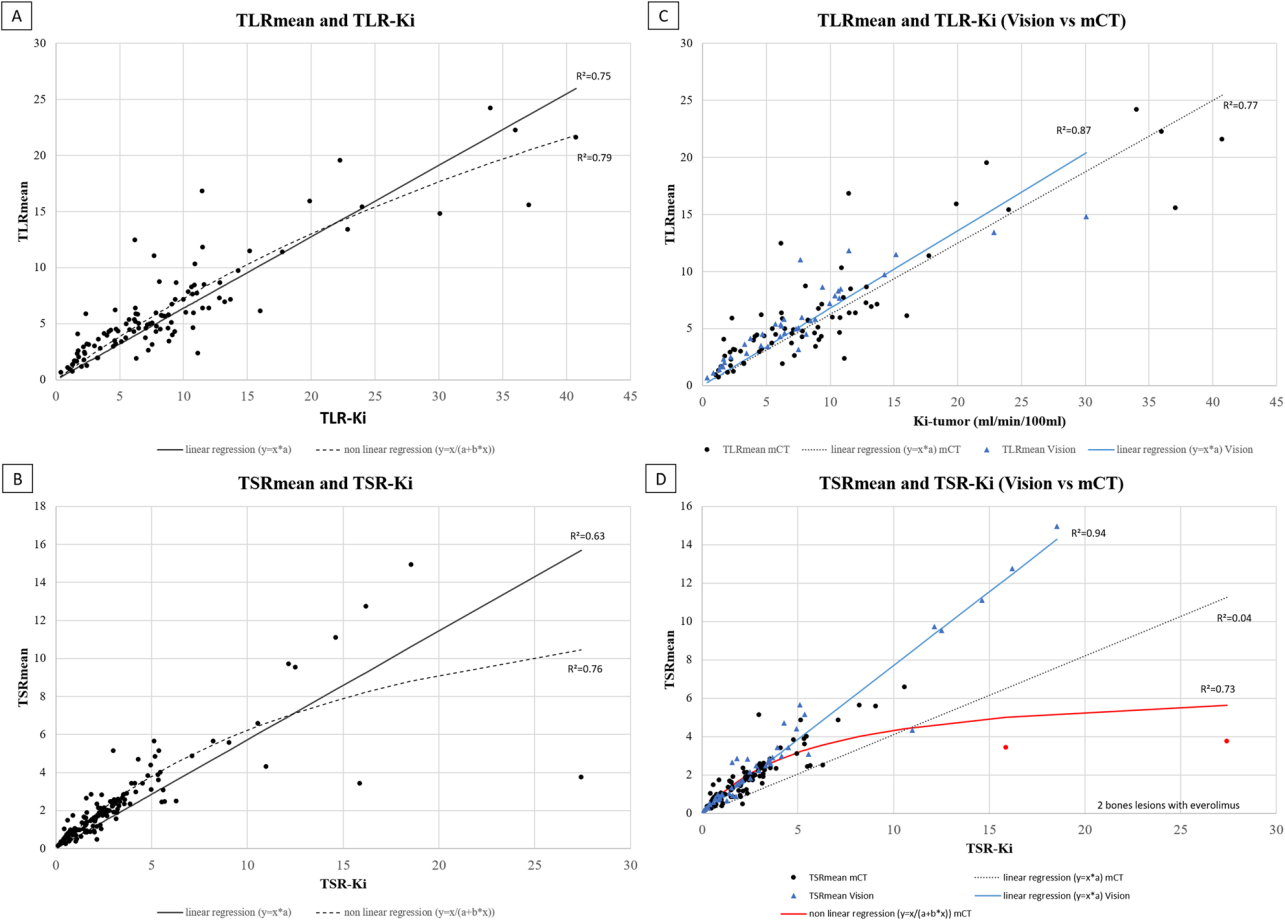
Correlation between TLRmean/TLR-Ki and TSRmean/TSR-Ki in the whole cohort and according to PET system. *Red circle correspond to the 2 bones lesions of patient 58.

Regarding TLR ratios, the relation between TLRmean and TLR-Ki was linear (R^2^ = 0.75). Using a non-linear regression does not appear to significantly improve the fit between the two metrics (R^2^ = 0.79) (Fig. 5A; Table 5). According to PET system, R^2^ were 0.87 and 0.77 in PET Vision and mCT system in linear regression, respectively (Fig. 5C).

Regarding TSR ratios, the relation between TSRmean and TSR-Ki was more tedious to fit. Using linear and non-linear regressions, R^2^ were 0.63 and 0.76, respectively (Fig. 5B; Table 5). According to PET system, non-linear regression appear more consistent describing the relation between TSRmean and TSR-Ki in mCT system (R^2^ = 0.73) while linear regression was more consistent describing the relation in PET Vision system (R^2^ = 0.94) (Fig. 5D). Due to these discrepancy results regarding the relation between TSRmean and TSR-Ki, we highlighted that one patient who underwent DWB acquisition on mCT system presented 5 lesions with low SUVmean values in comparison to Ki values, and particularly 2 bones lesions with very high Ki and TSR-Ki values (lesion 1, Ki = 184.0 ml/min/100 ml and TSR-Ki = 27.4; and lesion 2, Ki = 106.5 ml/min/100 ml and TSR-Ki = 15.9) comparing to lower SUVmean and TSRmean values (lesion 1, SUVmean = 65.9 and TSRmean = 3.7; and lesions 2, SUVmean = 60.2 and TSRmean = 3.4). TLR-Ki and TLR-mean were not available due to an important liver involvement in this patient.

### Static and dynamic parameters according to cohort characteristics : exploratory analysis

There was no significant difference in SUV and Ki-tumor values according to the tumor grade or the Ki67 result (p = NS). Patients with pancreatic NETs had significantly higher median SUV and Ki values than those with small intestine NETs (p < 0.001). The results are presented in Table 4.

According to SSA treatment, all median values of SUV and Ki parameters for liver and spleen uptake were statistically lower in patients treated with SSAs (p < 0.002). Regarding tumoral uptake, the median Ki-tumor value was significantly lower in patients treated with SSAs than the others (12.6 ml/min/100 ml [IQR, 8.4–17.6] vs 20.1 [IQR, 11.7–33], p = 0.027) while SUV-tumor values were not significantly lower in patients treated with SSAs (p = NS). The results are presented in Tables 3, 4.

#### Correlation analysis in patients with or without SSA treatment

**Figure 6:**
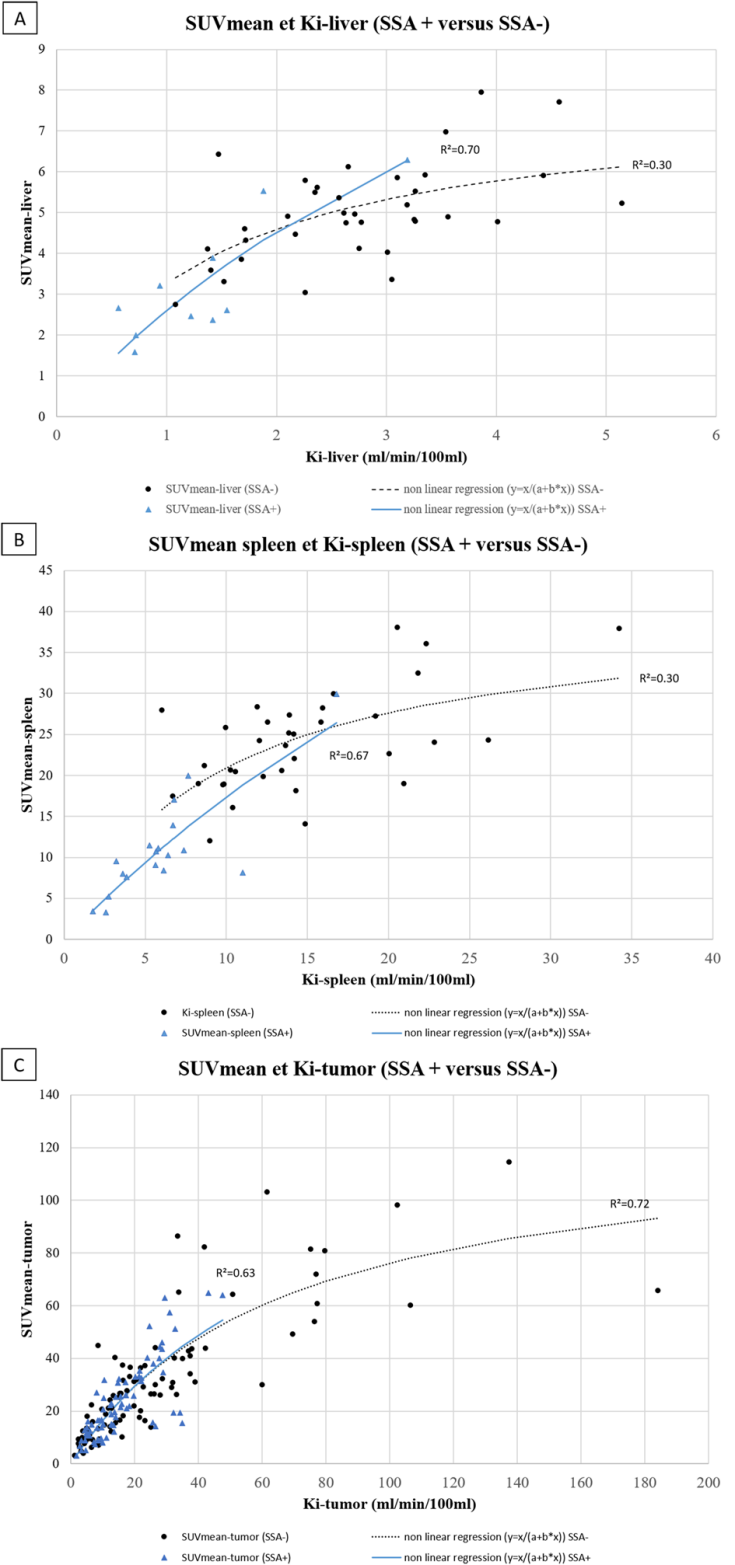
Correlation between SUVmean and Ki for physiological and tumoral uptakes according to SSA treatment.

Regarding physiological/tumor uptake and using non-linear regression, R^2^ were 0.70 and 0.30 in SSA+ and SSA− group for liver uptake, respectively (Fig. 6A). R^2^ were 0.67 and 0.30 in SSA+ and SSA− group for spleen uptake, respectively (Fig. 6B). R^2^ were 0.63 and 0.72 in SSA+ and SSA− group for tumor uptake respectively (Fig. 6C).

**Figure 7:**
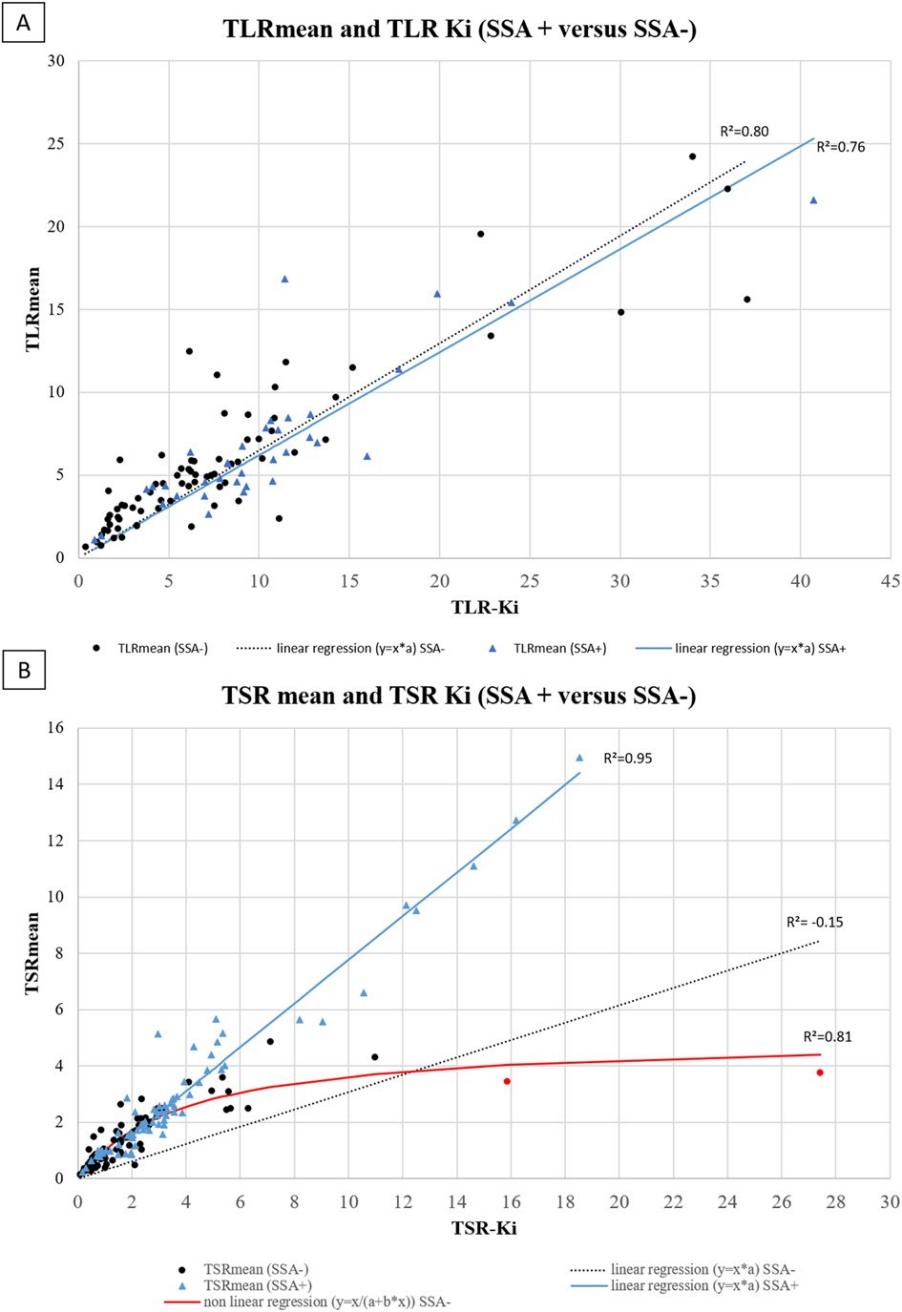
Correlation between TLRmean/TLR-Ki and TSRmean/TSR-Ki according to SSA treatment. *Red circle correspond to the 2 bones lesions of patient 58.

Regarding TLR ratios and using linear regression, R^2^ were 0.80 and 0.76 in SSA+ and SSA− group, respectively (Fig. 7A).

Regarding TSR ratios, non-linear regression appear more consistent describing the relation between TSRmean and TSR-Ki in patients with SSA- group (R^2^ = 0.81) while linear regression was more consistent describing the relation in SSA + group (R^2^ = 0.95) (Fig. 7B).

## Discussion

Our study is the first showing the feasibility of a DWB ^68^Ga-DOTATOC-PET/CT acquisition in a large cohort of 61 patients with WD-NETs. Our results highlight several important points allowing improving the comprehension of physiological and tumoral uptakes of ^68^Ga-DOTATOC in WD-NETs. We found that the Ki-liver was not influenced by the PET system used, unlike SUVmax and SUVmean suggesting that Ki may be a more robust parameter to assess liver uptake which is considered as a reference for images interpretation. Nevertheless, SUV and Ki parameters were not different for spleen and tumoral uptake between the two PET systems. Our correlation analysis between static and dynamic parameters suggested a non-linear relation between Ki and SUVmean in physiological and tumoral uptake and a linear relationship between TLRmean and TLR-Ki. We also found that these relationship seems not affected by the PET system on which the acquisition was performed.

In our study, we firstly assessed the physiological uptakes evolution in the liver and spleen organs in the whole cohort and according to the PET system. We showed that Ki values were not influenced by the PET system especially for liver uptake (p = 0.357); unlike SUVmean and SUVmax-liver that had higher values in a digital Vision system (p = 0.06 and p = 0.027, respectively). There was no statistical difference in SUVs and Ki values recorded on physiological spleen uptake within our analog and digital PET systems. So, these results suggested that the Ki may be a more robust and reproducible parameter especially for the liver uptake graduation that represent a reference in current practice for the quantification of SSTR expression by NETs. No other data are currently available in the literature on the comparison of DWB ^68^Ga-DOTATATE PET/CT acquisition according to different PET systems.

Then, we analysed the tumoral uptake and the correlation between SUVs and Ki values as already investigated in other studies, but in smaller cohorts of patients with divergent conclusions. In two studies conducted by the same team, authors concluded to a non-linear relationship between the SUV and Ki-tumor values recorded on both dynamic single-bed ^68^Ga-DOTATOC and ^68^Ga-DOTATATE PET/CT in patients with metastatic NET/NECs^13,15^ So, Velikyan et al. hypothesized that beyond a Ki > 20 ml/min/100 ml, SUV values did not increase. On the contrary, in a study including 20 patients with metastatic NETs who underwent a dynamic single-bed ^68^Ga-DOTATOC PET/CT acquisition before PRRT, Van Binnebeek et al. reported a linear relationship between Ki and SUVmean (R^2^ = 0.77). Nevertheless, the non-linear regression was not considered in the methodology^16^. In our study, we tested these two approachs to assess the relationship between static and dynamic parameters. First, we investigated the KSR according to the Ki value and showed that its median value increased significantly when Ki was higher than 20 ml/min/100 ml (p < 0.001). Then, we highlighted that a non-linear regression was more consistent to fit the relationship between SUVmean and Ki-tumor (R^2^ = 0.71 versus 0.40 using a linear regression), but also for the liver and spleen uptakes. Thereby, our findings are more in line with those found by Velikyan et al. and Ilan et al., even if our coefficient of determination was slightly lower but remaining high^13,15^. Performing the correlation analysis with only one lesion per patient (lesion with the highest Ki value), the results remained similar (supplemental Fig. 1). According to PET system, we highlighted that non-linear regression was also consistent to fit the relationship in both PET systems (R^2^ = 0.69 and R^2^ = 0.86 in analog and Vision system, respectively). These results support the hypothesis suggesting that the SUV only partially reflects the SSTR density in highly expressing SSTR tumors and may lead to an underestimation.

One hypothesis to explain this phenomenon of non-linear correlation is that the bioavailability of the peptide in plasma could be a limiting factor for radiotracer uptake in patients with high tumor burden and high expression of SSTR due to the “sink effect”^29^. In this situation, a rapid plasma clearance of the radiotracer is observed resulting in a low concentration of plasma activity. Then, the “sink effect” will result in apparent saturation of the SUV values^14^, unlike those of the Ki since plasma concentrations are taken into account for its calculation. Thus, Ilan et al. evaluated this concept on the assumption that the tumor-to-blood ratio (TBR) may be a better static parameter to assess the expression of SSTRs. Thereby, they showed an excellent correlation (R^2^ = 0.98) between tumor Ki and TBR values with a linear regression. The authors concluded that TBR better reflected the density of SSTR than the SUV alone and therefore could be the optimal measurement tool for the semi-quantitative assessment of tumor uptake of ^68^Ga-DOTATOC and ^68^Ga-DOTATATE^15^. In our method, we did not assess this TBR approach. However, we used the liver and spleen uptakes as references to calculate both static and dynamic TLR and TSR ratios. Regarding the correlation between TLRmean and TLR-Ki, the relationship appeared also linear (R^2^ = 0.75) in our cohort and the results were not difference in both PET systems. On the contrary, we found discordant results fitting the relationship between TSRs ratios with slightly higher correlation with a non-linear regression model than with the linear one (R^2^ = 0.76 versus 0.63, respectively). Using linear regression model according to PET system, we observed that TSRmean and TSR-Ki exhibited a strong correlation in the digital PET system subgroup (R^2^ = 0.94) but not in the mCT (R^2^ = 0.04). These discrepancy results are mainly explained by the profile of a 51-years-old female patient belonging to the mCT subgroup. Indeed, we observed very low TSRmean values but high corresponding TSR-Ki values in this patient, that have probably affected the correlation analysis (Fig. 5D). A potential explanation could be related to the associated treatment scheme. Indeed, this was the only patient treated with a targeted-therapy (everolimus) and without SSA treatment at the time of PET/CT acquisition. To our hypothesis, this treatment scheme may have a greater impact on the SUVmean-tumor than on the Ki-tumor value, as the associated anti-angiogenic effect may lead to a significant decrease in TSRmean but not in TSR-Ki. Another explanation is that the two lesions with the highest discrepancy results were bone metastases which could have a particular biodistribution pattern. Retrospectively, in excluding this patient from the correlation analysis, we finally found a significant higher coefficient of determination R^2^ with the linear regression model, increasing from 0.04 to 0.84 (supplementary Fig. 2). Consequently, we think that the interpretation of the SUV value in ^68^Ga-DOTATOC-PET/CT should be particularly careful and recommend further explorations in the comparison of static and dynamic quantitative approaches for the therapeutic response assessment in patients treated with everolimus. In summary, our results are supporting a non-linear relationship between SUVmean and Ki parameter and a linear relationship between TLRmean/TSRmean and TSR-Ki/TSRmean. We also found that these relationship seems not affected by the PET system used even if R^2^ appear slighty lower in analog versus digital PET system. That could be explained by their different intrinsic performances, the difference in our DWB acquisition protocol (i.e. step and shoot acquisition for mCT and continuous bed motion for Vision) and finally due to the difference between Ki calculation according to the two PET systems.

To assess if DWB acquisition can provide additional information in patients with WD-NETs, we also performed a preliminary analysis according to our cohort characteristics. Our results showed that SUV or Ki values were not statistically different in patients with G1 versus G2 NETs (or with Ki-67% ≥ 5% versus < 5%). These results are consistent with the literature. Indeed, some studies showed that SUVmax values were inversely correlated with Ki-67 expression and tumor grade when including both NETs and NECs (G1/G2 versus G3). However, this correlation does not exist when comparing only G1 and G2 NETs. In a retrospective study involving 126 patients with a gastroenteropancreatic NET undergoing a ^68^Ga-DOTATATE PET/CT, mean SUVmax values were significantly lower in G3 (12.8 ± 12.3) than in G1/G2 sub-groups (29.2 ± 28.6 and 26.0 ± 16.2, respectively; p < 0.05)^30^. In a study including 49 patients with a pancreatic NETs/NECs, Partelli et al. found median SUVmax values in ^68^Ga-DOTANOC-PET/CT even higher in the G2 than in the G1 sub-groups (53.5 and 31.5 respectively), while the median SUVmax value was 16.5 in the G3 pancreatic NETs subgroup^31^. In our cohort, the Ki-tumor values followed the same trends and therefore did not appear to be more discriminating. In our per-primary tumor origin analysis, patients with p-NET had significantly higher SUVs and Ki values compared to patients with si-NETs (p < 0.001) that was already suggested in the literature by Campana et al.^32^ and O'Toole et al.^33^.

According to SSA treatment, we found that both static and dynamic physiological uptake, decreased with the presence of an ongoing SSA treatment which is consistent with the literature data. Indeed, in a recent series of 21 patients with metastatic NETs undergoing ^68^Ga-DOTATATE PET/CT before and after SSA initiation, mean splenic and hepatic SUVmax decreased significantly between the two scans from 30.3 to 23.1 and 10.3 to 8.0 (p < 0.0001), respectively^34^. In our cohort, Ki-liver and Ki-spleen values appeared to follow the same trend with SUV parameters. Regarding our correlation analysis, we found that R^2^ values in SSA- patients were lower for liver and spleen uptakes using a non linear regression while R^2^ were comparable between SSA+ and SSA− groups for tumor uptake (R^2^ = 0.63 and 0.72), respectively. Regarding TLR ratios and using a linear regression, R^2^ were comparable between SSA+ and SSA− while we found a high discrepancy result for TSR ratios (R^2^ = 0.95 versus 0.15, respectively). As for mCT subgroup analysis, these results are also partially explained by the patient treated with everolimus (see above and supplementary Fig. 2).

To our knowledge, this is the first prospective study assessing a whole-body dynamic ^68^Ga-DOTATOC-PET/CT acquisition that makes possible to measure the Ki values for all the lesions included in the field of view. This offers interesting perspectives in terms of diagnostic, prognostic and therapeutic evaluation in NETs, which are tumors with a high prevalence of metastatic disease at the time of diagnosis.

Our study presents several limitations. Firstly, our cohort is monocentric and our population is heterogeneous, including patients with localized and metastatic NETs from different primary origins and with different ongoing treatments. Secondly, we did not perform arterial blood samples to adjust our input function. Nevertheless, several study showed that the input function can be determinated non invasively within different validated methods^16,35^. To our mind, DWB acquisition needs to remain as minimally invasive as possible to be implemented in clinical routine. Finally, we used the left ventricular VOI in our study to assess the input function. Using the descending thoracic aorta to generate the IF as previously reported^13–15^ could have been a better option. Thus, we retrospectively performed a supplementary work on 17 other control patients included in the GAPETNET study, who underwent the DWB acquisition on the PET Vision system. And we did not found any significant difference in Ki values whatever the IF estimation method (left ventricular vs descending aorta VOI) and the Patlak analysis (automated vs graphical) used (supplemental Table 1).

In conclusion, our study is the first study showing the feasibility of a DWB acquisition in ^68^Ga-DOTATOC-PET/CT in both analog and digital PET systems. The comparison of the parameters obtained on the two PET systems suggests that Ki may be a more robust parameter especially for assessing liver uptake. Our findings confirm that the correlation between the tumoral SUVmean and Ki values follows a non-linear relationship as already described in previous studies. Our exploratory analysis suggests that combining SUV-derived with Ki-derived metrics could lead to a better characterization of the physiological and tumoral tracer uptake, providing in-vivo additional quantitative value in the characterization of SSTR expression in WD-NETs. Future results of our prospective prognostic trial will surely allow us to better understand the behavior of the WD-NETs and their biological environment with Ki values.

## Supplementary Information

Supplementary Information 1.

Supplementary Information 2.

Supplementary Information 3.

Supplementary Information 4.

## Data Availability

The datasets analysed during the current study are available from the corresponding author on reasonable request.

## Abbreviations

18FDG: 18-Fluorodesoxyglucose
68Ga-DOTA-SST: Gallium-68 DOTA-conjugated somatostatin receptor-targeting peptide
AUC: Area under the curve
DCB: Dynamic cardiac-bed
DWB: Dynamic whole-body
IF: Input function
Ki: Net uptake rate
KSR: Ki-tumor to SUVmean-tumor ratio
NECs: Neuroendocrine carcinomas
NETs: Neuroendocrine tumors
PET/CT: Positron-emission tomography computed tomography
PRRT: Peptide-receptor radionuclide therapy
ROI: Region of interest
SSA: Somatostatin analogs
SSTR: Somatostatin receptors
SUV: Standardized uptake value
TAC: Time-activity curve
TBR: Tumor-to-blood pool ratio
TLR-Ki: Ki-tumor to Ki-liver ratio
TLRmean: SUVmean-tumor to SUVmean-liver ratio
TSR-Ki: Ki-tumor to Ki-spleen ratio
TSRmean: SUVmean-tumor to SUVmean-spleen ratio
VOI: Volume of interest
WD-NETs: Well-differentiated NETs
