# Supplementary material for: Prospective study of dynamic whole-body 68Ga-DOTATOC-PET/CT acquisition in patients with well-differentiated neuroendocrine tumors
Source: Sci Rep. 2021 Mar 1;11:4727. doi: 10.1038/s41598-021-83965-9 (PMC7921579; doi:10.1038/s41598-021-83965-9)
Supplement: Supplementary file 1 — Supplementary Information 1. [file 41598_2021_83965_MOESM1_ESM.docx]

**Supplementary figure 1 :** Correlation between SUVmean/Ki-tumor, TLRmean/TLR-Ki and TSRmean/TSR-Ki using one lesion per patient (lesion with highest Ki value)

**Supplementary figure 2 :** Correlation between TSRmean and TSR-Ki in mCT and SSA subgroups excluding patient 58.
