# Supplementary figures and images for: Prospective study of dynamic whole-body 68Ga-DOTATOC-PET/CT acquisition in patients with well-differentiated neuroendocrine tumors
Source: Sci Rep. 2021 Mar 1;11:4727. doi: 10.1038/s41598-021-83965-9 (PMC7921579; doi:10.1038/s41598-021-83965-9)

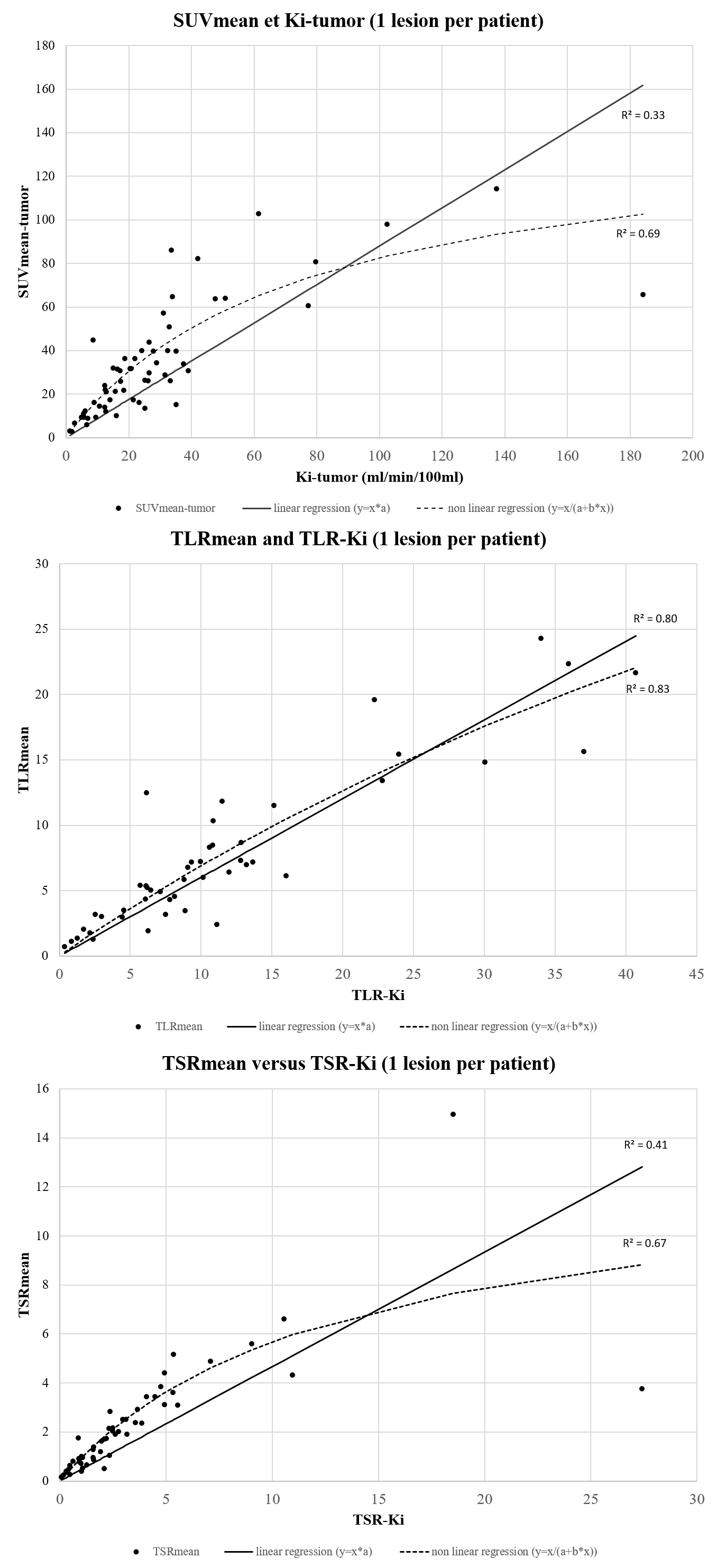

Supplement: Supplementary file 2 — Supplementary Information 2. [file 41598_2021_83965_MOESM2_ESM.png]

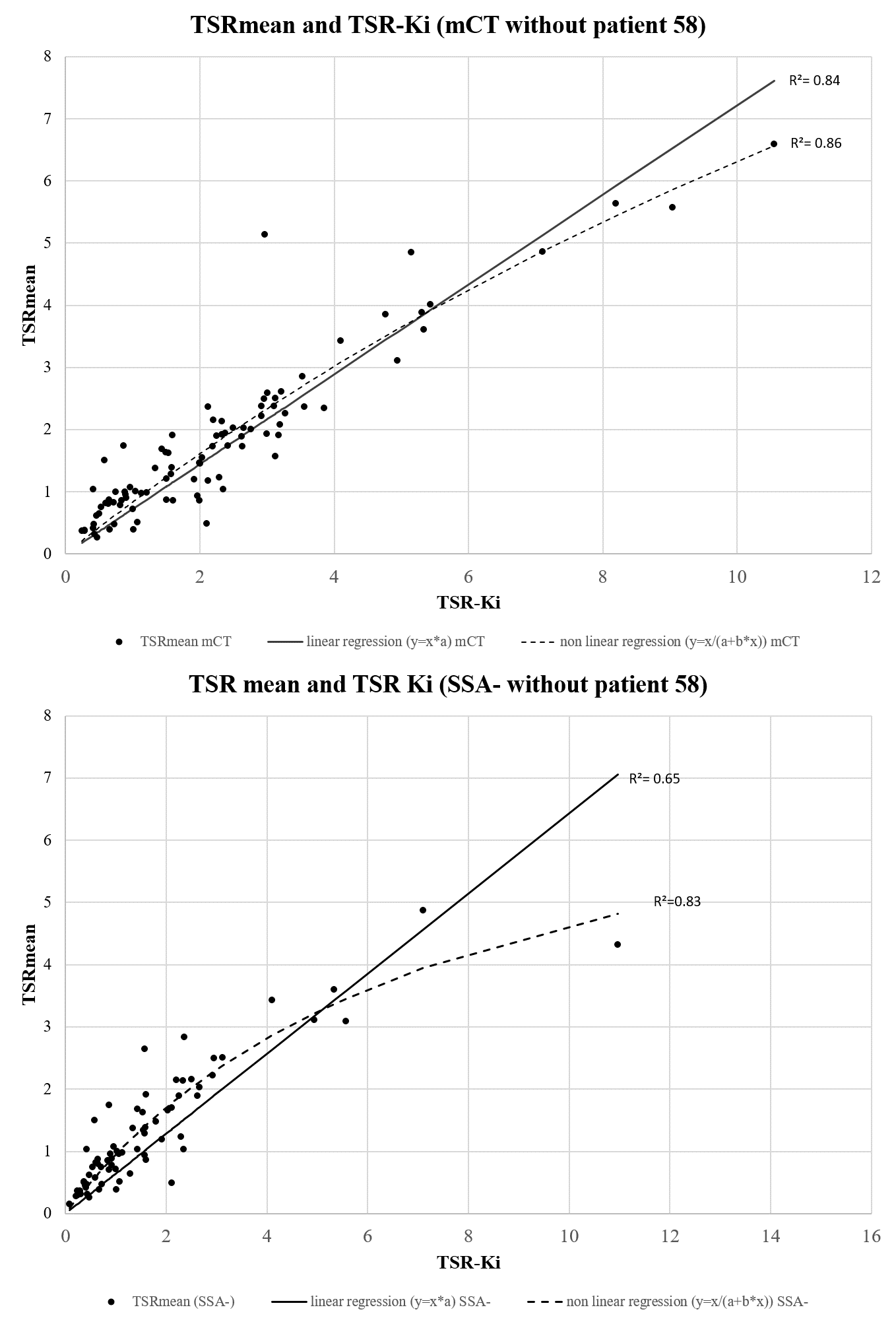

Supplement: Supplementary file 3 — Supplementary Information 3. [file 41598_2021_83965_MOESM3_ESM.png]
