# Supplementary material for: Prospective study of dynamic whole-body 68Ga-DOTATOC-PET/CT acquisition in patients with well-differentiated neuroendocrine tumors
Source: Sci Rep. 2021 Mar 1;11:4727. doi: 10.1038/s41598-021-83965-9 (PMC7921579; doi:10.1038/s41598-021-83965-9)
Supplement: Supplementary file 4 — Supplementary Information 4. [file 41598_2021_83965_MOESM4_ESM.docx]

|  | (a) versus (b) | (a) versus (c) | (b) versus (c) |
| --- | --- | --- | --- |
| Mean difference (%) |  |  |  |
| Ki-liver | 3.96 | 2.89 | 0.24 |
| Ki-spleen | 0.11 | -2.67 | -3.59 |
| Bland-altman (bias [CI95%]) |  |  |  |
| Ki-liver | 0.01 [-0.54; 0.56] | 0.06 [-0.46 ; 0.58] | 0.05 [-0.07 ; 0.17] |
| Ki-spleen | - 0.18 [-0.96; 0.60] | 0.34 [-2.17 ; 2.84] | 0.51 [-0.02 ; 10.47] |
| Intraclass correlation coefficient |  |  |  |
| Ki-liver | 0.976 | 0.980 | 0.983 |
| Ki-spleen | 0.978 | 0.984 | 0.988 |

**Supplemental table 1:** Robustness of Ki parameters according to the calculation method used in 17 patients who underwent the DWB acquisition on PET Vision system. ; a) Patlak graphical analysis with IF generated on a VOI centered on the left ventricle or b) Patlak graphical analysis with IF generated on a VOI centered on the descending aorta or c) Automated Patlak Reconstruction with IF generated on a VOI centered on the left ventricle
